# Effect of Root Canal Sealers on Bond Strength of Fiber Posts to Root Dentin Cemented after one Week or six Months

**DOI:** 10.22037/iej.v13i1.17998

**Published:** 2018

**Authors:** Lucas Ruiz, Giovana Mongruel Gomes, Bruna Bittencourt, Fabrício Rutz da Silva, Osnara Maria Mongruel Gomes, Julio Cezar Chidoski Filho, Abraham Lincoln Calixto

**Affiliations:** a *Department of Dentistry, School of Dentistry, Universidade Estadual de Ponta Grossa. Ponta Grossa, Paraná, Brazil*

**Keywords:** Cementation, Dentin, Push-out Bond Strength, Resin Cements, Zinc Oxide Eugenol Cement

## Abstract

Eugenol-based root canal sealers (RCS) have been widely used by clinicians; however, their effect on resinous materials is still questionable. The objective of this study was to evaluate the influence of RCS at 1 week and 6 months’ post obturation on the bond strength (BS) of glass fiber posts (GFP) to root dentin, using conventional and self-adhesive cementation systems (CS). The roots of 56 extracted human canines, were divided in eight groups (*n*=7) according to the combination of the following factors: RCS (with or without eugenol-Endofill and Sealer 26, respectively), storage period post obturation and prior GFP cementation (1 week and 6 months) and cementation systems (Variolink II - conventional resin cement or RelyX U200-self-adhesive resin cement). After one week, the specimens were transversely sectioned into six 1-mm-thick disks and were subjected to the push out BS test. The data were subjected to 3-way ANOVA and Tukey’s tests (*α*=0.05). The BS were not affected by the RCS, neither the CS (*P*>0.05). Just the period post obturation showed statistically significant differences (*P* 0.05), where the GFP cemented 6 months after the endodontic treatment showed higher values than those cemented 1 week after it.

## Introduction

Endodontically treated teeth with few remaining tooth structure require the use of intracanal posts associated with resin cores to retain the final restoration [1-3]. About the failures which may occur with the use of intracanal posts, root fractures can be named as the worst one [4]. In order to reduce these risks, glass fiber posts have been widely used as they form a mechanically homogeneous structural complex between the post, resin cement, composite and root dentin; thereby, reducing the risk of radicular fracture due to stress absorption generated by masticatory forces [5, 6].

Like all bonding process, the successful use of glass fiber post is associated with bond quality of post-cement-dentin, to ensure an adequate adhesive interface [7]. One of the factors which may affect the adhesion between resin cements and root dentin is the root canal sealer (RCS) composition [8]. Eugenol-based RCS are widely used by clinicians [9]. Their effect over resinous materials is still questionable. While studies report lower bond strength values with the prior use of eugenol-based RCS [8, 10], other demonstrated no negative effect of these materials over glass fiber posts cementation [11-13]. 

Not just the sealer may affect the intra-radicular adhesion; also, the time elapsed between endodontic treatment and adhesive cementation [9, 14-16]. A recent study verified that longer periods from the root canal obturation until post cementation had a negative effect on the bond strength [16], possibly due to greater penetration of eugenol within the dentinal tubules [15], while a systematic review concluded that eugenol-based sealer reduces the immediate bond strength of fiber posts with resin cement [17]. Otherwise, studies showed no influence of eugenol-based RCS after 24 h, 7 days [18] and 3 months [19] in the resin cements microleakage.

The cementation strategy also influences post adhesion to root dentin. Conventional cementation systems (CS) significantly reduced the eugenol amount within the dentinal tubules, which is removed in the acid etching step with phosphoric acid [1, 19]. It is worth mentioning that most of the studies have evaluated the interaction of eugenol-based RCS *versus* conventional RCS. Researches which evaluated the new generation of self-adhesive resin cements reported conflicting results [8, 10, 20]. Little is known about their adhesive behavior, as the smear layer is incorporated into the hybrid layer [21], and consequently a greater eugenol amount would remain within the dentinal tubules. 

Considering which was mentioned, the objective of this study was to evaluate the influence of eugenol and non-eugenol based RCS in the bond strength of glass fiber posts to root dentin, using a conventional and a self-adhesive CS, after two storage periods (SP). The null hypotheses were: 1) root canal sealers, 2) cementation systems or 3) the storage periods would not influence the bond strength between glass fiber post and root dentin.

## Materials and Methods

The Ethics Committee of the Local University approved this study. Fifty-six extracted human maxillary canines, with single canals, were stored in distilled water at 4^º^C and used within 6 months after extraction (ISO 11405:2003). The inclusion criteria were: absence of restoration, caries or root cracks, absence of previous endodontic treatments, posts or crowns, absence of severe root curvatures and a root length of 15±1 mm, measured from the cemento-enamel junction (CEJ). 

***Specimen preparation***

Teeth were transversally sectioned 1 mm above the CEJ using a low diamond saw (Isomet 1000; Buehler, Lake Bluff, IL, USA) under constant irrigation. After it, radiographs were performed from facial and proximal views to ensure the presence of a single canal. Endodontic access was made using a tapered fissure bur with a high-speed handpiece and water spray. Apical limit of root treatment was determined 1 mm above the apical foramen. A crown-down technique was used for instrumentation with K-files (Dentsply, Petrópolis, RJ, Brazil). Apical enlargement was performed to size 40 with 0.02 taper. Irrigation procedures were accomplished by using 2 mL of 1.0% sodium hypochlorite (NaOCl) for each file used. To remove the smear layer, all canals were irrigated with 3 mL of 17% EDTA over 2 min followed by 2 mL 1.0% NaOCl over 1 min and in the last instrumentation the canals were irrigated with 10 mL of distilled water and dried with sterile paper points (Dentsply, Petrópolis, RJ, Brazil) [4, 22, 23].

The roots were randomly divided into eight groups (*n*=7), according to the combination of the following factors: root canal sealer (with or without eugenol), storage period post obturation and prior GFP cementation (1 week or 6 months) and cementation system (adhesive system and conventional resin cement or self-adhesive resin cement).

***Root canal obturation***

The root canals were filled with the corresponding root canal sealers (RCS) -an eugenol-based sealer Endofill (Dentsply, Petrópolis, RJ, Brazil) and a calcium hydroxide-based: Sealer 26 (Dentsply, Petrópolis, RJ, Brazil) according to their manufacturers’ instructions and tapered with gutta-percha points using the vertical warm condensation technique. To ensure adequate filling of the root canals, radiographs were performed from facial and proximal views. The root access was temporarily filled with an adhesive system (Âmbar; FGM, Joinvile, SC, Brazil) and a composite resin (Opallis; FGM, Joinvile, Santa Catarina, Brazil). 

***Storage period post obturation and prior GFP cementation***

In the 1 week storage period (SP) groups, after the obturation with the RCS and gutta-percha, the roots were stored at 37±2^º^C in 100% humidity for 7 days; and in the 6 months SP groups, the roots were stored in the same condition for 180 days. 

Just after these periods, the root canals were prepared to receive the glass fiber posts (Whitepost DC#1, FGM, Joinville, SC, Brazil). For this purpose, the gutta-percha was removed from the root canals firstly using Gates Glidden burs #2 to 4, (Dentsply, Petrópolis, RJ, Brazil), followed by the carbide burs corresponding of the post Whitepost DC #1, leaving 4 mm of the apical seal. The root canals were then irrigated with 10 mL of distilled water, and then dried over 5 sec with an air stream and one paper point # 80 (Dentsply, Petrópolis, RJ, Brazil). The working length of the post space was 11 mm for all teeth, to maintain the apical 4-mm apical filling. All specimens were prepared by one operator in a standardized procedure. 

***Cementation procedures***

Before cementation, the glass fiber posts were horizontally sectioned at the coronal region with a water-cooled diamond cutting instrument to reduce the post length to 14 mm. While 11 mm were cemented inside the root canal the coronal 3 mm served as a guide to standardize the distance of the light curing device from the cervical root area. 

The post cementation procedures were performed according to the different cementation systems (CS) tested: a total-etch adhesive system (Excite DSC; Ivoclar-Vivadent, Schaan, Liechtenstein) and conventional resin cement (Variolink II; Ivoclar-Vivadent, Schaan, Liechtenstein), and a self-adhesive resin cement (RelyX U200; 3M ESPE, Seefeld, Germany). 

The posts were tried in, cleaned with 70% alcohol for 5 sec and cemented in accordance with the manufacturer’s instructions for each cementation system described in Table 1. The resin cement was inserted into the root canal space through an insulin syringe No. 0 (BD; São Paulo, SP, Brazil) with a #40 pink needle (Injex; Ourinhos, SP, Brazil). Then, the glass fiber post was positioned into place. A LED light curing device (L.E.Demetron I; Kerr Corp., Orange, CA, USA) with a power density of 800 mW/cm^2^ was used for curing purposes. After the post luting procedures, all samples were stored in water at 37^º^C for one week [4].

***Sample preparation***

The roots were embedded in polyvinyl chloride (PVC) tubes using acrylic resin (Duralay; Reliance Dental, Alsip, IL, USA), and the portion of each root containing the bonded fiber post was sectioned perpendicular to the long axis into six serial slices, 1 mm thick, using the Isomet 1000 (Buehler, Lake Bluff, IL, USA) with a water-cooled diamond saw. The coronal side of each slice was identified and its thickness measured with a digital caliper -accuracy of 0.01 mm (Mitutoyo Corp., Aurora, IL, USA). The slices were also photographed on both sides, with an optical microscope (Olympus, Tokyo, Japan) under a 40× magnification in order to measure the coronal and apical diameters of the posts, with the purpose of calculating their individual bonding area. This measurement was performed with UTHSCSA Image Tool 3.0 software (Department of Dental Diagnostic Science, University of Texas Health Science Center, San Antonio, TX, USA) [4, 22, 23].

***Push-out bond strength test***

Each specimen (slices) was subjected to a push-out test using a universal loading device (AG-I; Shimadzu Autograph, Barueri, Tokyo, Japan) at a crosshead speed of 0.5 mm/min with the load applied in the apical-coronal direction until the post was dislodged. Care was taken to center the push-out pin on the center of the post surface without stressing the surrounding post space walls [4, 23-25]. Different sizes of punch pins were used to match the diameter of the post at the different root canal thirds. Three different sizes of punch pins were selected, one representative for each root canal region: cervical (1.4 mm), medium (1.0 mm) and apical (0.6 mm).

The maximum failure load was recorded in Newton (N) and converted into MPa by dividing the applied load by the bonded area (S_L_). The latter, being the lateral surface of a truncated cone, was calculated by the formula: S_L_=π(*R*+*r*) [(*h*^2^+(*R*–*r*)^2^]^0.5^, where *π*=3.14, *R*=coronal post radius, *r*=apical post radius, and *h*=root slice thickness. 

***Failure modes analysis***

After push-out evaluation, the failure modes of all specimens were evaluated under a stereomicroscope (40× magnification). The failure modes were classified according to the following criteria: Type I, adhesive failure between dentin and resin cement; type II, adhesive failure between resin cement and post; type III, cohesive failure within dentin; type IV, cohesive failure within cement; type V, cohesive failure within post; type VI, mixed failures. Two independent and calibrated operators analyzed each fractured specimen. If any disagreement occurred between the evaluators, a consensus had to be obtained [2, 26].

After the classification of failure modes, scanning electron micrographs of each representative fracture pattern were obtained, so the specimens were processed for scanning electron microscopy (SEM) evaluation. The slices were rinsed in a 95% alcohol solution for 1 min, air-dried, mounted on a metal stub and sputter-coated with gold-palladium (Polaron SC7620; Quorum Technologies Ltd., Newhaven, UK) for 5 min at 10 mA. After this, the specimens were examined by SEM (JSM 6360LV; Jeol Ltd., Tokyo, Japan) at a 15-kV accelerating voltage under different magnifications (40× and 200×) and photographs were taken. 

***Statistical analysis***

The experimental unit for all properties evaluated was the root. Therefore, an average of the values collected from the slices of each root canal was obtained per tooth for statistical purposes. The data obtained from bond strength were subjected to three-way ANOVA (root canal sealer *vs* storage period *vs* cementation system) and Tukey’s test (alpha=5%). The difference in the failure patterns were analyzed by *chi*-square test. The Sigma Plot 11 software (Systat Software, San Jose, CA, USA) was used for statistical analysis.

## Results

The mean±standard deviation (mean±SD) of bond strength values, in MPa, of the different experimental groups are shown in Table 2. 

**Table 1:** Bonding procedures

**Cementation system/Manufacturer**	**Composition**	**Application mode**
**Excite DSC/Ivoclar-** **Vivadent + Variolink II/ Ivoclar-Vivadent**	Excite DSC: Phosphonic acid acrylate, hydroxyethyl methacrylate dimethacrylates, highly dispersed silicon dioxide, ethanol, catalysts and stabilizersVariolink II: Bis-GMA, UDMA, TEGDMA, barium glass filler, ytterbium trifluoride, mixed oxides, Ba-Al fluoro-silicate glass, catalysts and stabilizers	1. Application of 37% phosphoric acid gel for 15 sec in the canal; 2. Rinsing with water for 15 sec followed by air drying; 3. Remove excess moisture with a paper point leaving the dentin slightly moist; 4. Apply two coats of the adhesive system in canal and remove excess with a paper point; 5. Dispense cement onto a mixing pad and mix for 10 sec; 6. Apply cement in and around canal; 7. Place a thin layer of mixed cement on post and seat the post; 8. Remove excess cement while holding post in place; 9. Light-polymerize for 20 s from an occlusal direction.
**RelyX U200/3M ESPE**	Base: mixture of mono-, di- and tri-glycerol esters of phosphoric acid dimethacrylate, TEGDMA, silane-treated glass, silane treated silica, glass, sodium persulfate, tert-butyl peroxy- 3,5,5 trimethylhexanoate. Catalyst: substituted dimethacrylate, 1,12-dodecane dimethacrylate, silane-treated glass, silane-treated silica, calcium hydroxide, calcium salt of 1-benzyl-5-phenyl-barbic-acid, sodium p-toluenesulfinate	1. Irrigation the canals with 2.5% NaOCl and with distilled water; 2. Remove excess moisture with a paper point; 3. Dispense cement onto a mixing pad and mix for 20 sec; 4. Apply cement in and around the canal; 5. Place a thin layer of mixed cement on post and seat the post; 6. Remove excess cement while holding post in place; 7. Light-polymerize for 20 sec from an occlusal direction.

* *Bis-GMA, bisphenol A diglycidyl methacrylate; UDMA, uretane dimethacrylate; TEGDMA, triethylene glycol dimethacrylate*

**Table 2:** Mean (SD) of bond strength values, in Mpa, of the different experimental groups according to the root canal sealers (Endofill and Sealer 26), cementation systems (Variolink II and RelyX U200), and storage period (1 week and 6 months) post obturation.

**Experimental Groups**	**Storage period post obturation**	
	**1 Week**	**6 Months**
**Endofill + Variolink II**	10.38 (1.71)	12.39 (3.09)
**Endofill + RelyX U200**	11.75 (2.03)	12.83 (0.94)
**Sealer 26 + Variolink II**	12.16 (1.73)	12.37 (1.29)
**Sealer 26 + RelyX U200**	11.34 (1.14)	13.77 (2.47)
**Main factor**	11.41 (0.76) ^A^	12.84(0.65) ^B^

**Table 3:** Absolute distribution of the failure mode (in %) of the different experimental groups according to the root canal sealers (Endofill and Sealer 26), cementation systems (Variolink II and RelyX U200), and storage period (1 week and 6 months) post obturation*

**Experimental Groups**	**Storage period post obturation**	
	1 Week	6 Months
**Endofill + Variolink II**	67/5/0/2/2/24	71/5/0/5/2/17
**Endofill + RelyX U200**	52/6/0/7/7/28	57/2/0/7/5/29
**Sealer 26 + Variolink II**	72/7/0/2/0/19	74/7/0/5/0/14
**Sealer 26 + RelyX U200**	60/5/0/2/7/26	64/8/0/2/2/24

* *Type I: adhesive between* *dentin and resin cement; type II: adhesive between resin* *cement and post; type III: cohesive within dentin; type* *IV: cohesive within cement; type V: cohesive within post;* *type VI: mixed failures*

**Figure 1:**
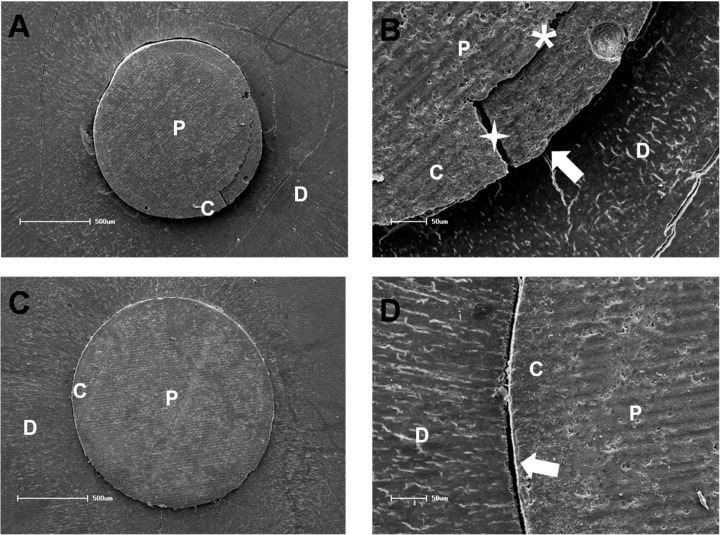
*A)* Scanning electron micrographs of representative fracture patterns. A mixed failure mode can be seen in low and high magnification; *B)* one can observe the adhesive failure between the cement and the dentin interface (arrow) along with a cohesive failure within cement (star) and an adhesive failure between the cement and the post (asterisk); *C)* An adhesive failure mode can be seen in low and high magnification; *D)* The failure occurred between the cement and dentin (arrow) (Abbreviations: D-dentin; P-post; C-cement)

Three-way analysis of variance detected significant differences just between the storage period groups after the endodontic treatment (*P*=0.004). The third-order (cross-product) interaction between the independent variables (RCS *vs* CS *vs* SP) was not significant (*P*=0.107); as well as the second- order interaction (RCS *vs* CS, *P*=0.528), (RCS *vs SP, P=0.817), (CS vs SP, P=0.501) were also not significant.*

The distribution of the failure modes is showed in Table 3. The most predominant failure pattern was Type I (between resin cement and dentin), followed by Type VI (mixed failures). Only few Type IV (adhesive failures between the cement and post), and Type V failures (cohesive fractures in cement and post, respectively) were observed. No Type III failures (cohesive fracture in dentin) were observed (Table 3). Representative scanning electron microscopy (SEM) images of the predominant failures are illustrated in Figure 1. No significant difference was found between the different experimental groups (*chi*-square test: *P*>0.05).

## Discussion

The interaction between the resinous material and the eugenol-based RCS may be explained by its setting reaction. The RCS powder (zinc oxide) and liquid (eugenol) handling generates a zinc eugenolate matrix formed by a chelating reaction. However, in the presence of dentinal fluid, this reaction may become reversible and eugenol releases from the matrix [27], influencing the polymerization reaction of the resin cements, which, in turn, may decrease bond strength values between root dentin and glass fiber post [8, 10]. The hydroxyl group of eugenol molecule tends to protonize the free radicals formed during the polymerization reaction of the resin-based materials, retarding the polymer formation [28], and may decrease bond strength values between dentin and the resinous material [29], since eugenol is still present within the dentinal tubules for 28 days after treatment [14].

The first and second null hypotheses were accepted. According to other studies [11, 13, 19], the eugenol-based RCS were not able to negatively influence the adhesion of glass fiber posts cemented either with conventional or self-adhesive resin cements. Controversial results were found by Cecchin *et al.* [8]. These differences may be explained by the difficulty of debris removal within the root canals, both with manual or rotary instruments [13]. The authors concluded that the root canal cleaning and preparation are more important factors than the actual choice of an endodontic sealer. 

The third null hypothesis was rejected, as the time elapsed between root canal sealing and post cementation significantly affected the bond strength, which showed better results after 6 months of endodontic obturation. Similar results were found in the literature [9]. A possible explanation is that eugenol diffusion occurs in the first 24 h, and slowly decreases after 7 days. However, Hagge *et al.* [15] reported different results, as they hypothesized that a longer time elapsed since the root canal filling until post cementation has a negative influence in the adhesion between resin cement and root dentin, probably because higher penetration of eugenol occurs within the dentinal tubules. However, other study [19] demonstrated no significant differences between the post cementation period (after 24 h and 3 months), regardless of the RCS used. These authors used only a conventional CS, and eugenol inside the dentinal tubules would be removed by the chemo-mechanical preparation and acid etching of the root canal done before the post cementation; speculating that eugenol would not influence microleakage through time. 

With respect to the self-adhesive resin cement, there was no influence of RCS type in bond strength between the post and root dentin. Other authors found that RelyX Unicem showed higher values compared to Variolink II and ParaCore cements with the previous use of an eugenol-based RCS [11, 16]. However, Cecchin *et al.* [8] found that Endofill negatively influenced bond strength values of RelyX Unicem, compared to a calcium-hydroxide-based RCS. 

Regarding failure modes, there was no statistically significant difference between the experimental groups. The predominant failure pattern was adhesive (between resin cement and dentin) followed by mixed, which agrees with the results of some authors [2]. This is due to the complexity of adhesion on the root dentin as well as their biological and structural characteristics.

Some questions regarding the effect of eugenol-contained CS remains uncertain, related to eugenol concentration, penetration and length of time within the dentinal tubules. Further researches are still required, as these techniques and materials belong to everyday clinical practice and may determine the success or failure of the rehabilitation treatment.

In the present investigation, the push-out bond strength test was used to evaluate the strength of the bonding between the fiber post to the root canal as this test simulates closely the clinical conditions. This test provides a better estimation of the bonding strength than the conventional shear test because the fracture occurs parallel to the dentin-bonding interface, which makes it a true shear test [24].

## Conclusion

According to the results, the bond strength values were not affected by the root canal sealer, neither the cementation system; however, the bond strength between glass fiber post and root dentin was higher after six months than one week post-obturation of the root canals.
